# Evaluation and Comparison of Mechanical Properties of Heat Polymerized Acrylic Resin After Reinforcement of Different Fibers in Different Patterns: An In Vitro Study

**DOI:** 10.7759/cureus.39564

**Published:** 2023-05-27

**Authors:** Karvika Nayak, Tripty D Rahangdale, Saurabh Shrivastava, Prabha S Newaskar, Nishi Mishra, Syed Mohammed Noorani

**Affiliations:** 1 Department of Prosthodontics and Crown and Bridge, Mansarovar Dental College, Hospital and Research Centre, Bhopal, IND; 2 Department of Prosthodontics and Crown and Bridge, Rural Dental College, Pravara Institute of Medical Sciences, Loni, IND; 3 Department of Oral Radiology and Medicine, Mansarovar Dental College, Hospital and Research Centre, Bhopal, IND

**Keywords:** bidirectional reinforcement, unidirectional reinforcement, polymethyl meth-acrylate, glass fibers, flexural strength, basalt fibers

## Abstract

Introduction: Most denture fractures occur within the mouth due to resin flexural fatigue. For example, the deep labial notch at the high labial frenum causes denture breakage, as can deep scratches and generated processing stresses. The rising cost of annual prosthetic repairs is evidence that the problem of total denture fracture has not been solved. The purpose of this investigation was to evaluate the relative improvement in flexural strength between heat-cured polymethyl methacrylate (PMMA) resin reinforced with glass fibers (GF) and basalt fibers (BF) of varied orientations.

Material and methods: A total of 150 heat-cured acrylic resin specimens of 65x10x3 mm dimension were prepared, 30 of which were left unreinforced (Group A), 30 of which were reinforced with GF in transverse pattern (Group B), 30 of which were reinforced with GF in meshwork pattern (Group C), 30 of which were reinforced with BF in transverse pattern (Group D), and 30 of which were reinforced with BF in meshwork pattern (Group E). All of the samples were put through flexural strength testing on the universal testing machine. One-way ANOVA and the Tukey-Kramer various correlation test (= 0.05) were used in SPSS for Windows to look at the facts.

Results: The mean flexural strength for Group A was 46.26±2.26 MPa, 64.98±1.53 MPa for Group B, 76.45±2.67 MPa for Group C, 54.22±2.24 MPa for Group D, and 59.02±2.38 MPa for Group E. Flexural strength was impacted by both the kind of BF and GF reinforcement (F = 768.316, P = 0.001).

Conclusion: Within the limitation of the current research, BF reinforcement outperforms GF reinforcement and unreinforced heat-cured acrylic resin in terms of flexural strength.

## Introduction

Polymethyl methacrylate (PMMA) is the material of choice for removable prostheses because of its superior working properties, ease of processing, exact fitting, oral stability, and great aesthetics [1]. Despite these great qualities, there is still scope for growth, most notably in terms of strength [2]. PMMA is put through a variety of stresses, including compression, tensile, shear, and impact, while it is in use. Fracture is a common concern with removable dentures due to the risk of them falling accidentally, experiencing repeated masticatory stresses, and having stress concentrations at deep frenal notches [3]. The majority of denture fractures happen inside the mouth as a result of resin flexural fatigue [4]. For instance, a deep labial notch at the high labial frenum can cause denture breakage. Deep scratches and processing stresses can also cause denture breakage. The fact that the problem of total denture fracture has not been solved despite the rising annual costs of prosthetic repair [4] is evidence of this. Flexural fatigue failure from cyclic deformation can be identified by a maxillary or mandibular full denture that has a midline fracture [5].

Various fiber reinforcements have been proposed as remedies, recommended for strengthening acrylic resin [4-17], nanoparticles [18-32], and nanotubes [33-37]. Various studies have been conducted on carbon, aramid, woven polyethylene, and glass fibers (GF), with varying degrees of success. Strength was increased in PMMA thanks to the addition of these fibers, but polishing and appearance were negatively affected [7]. The flexural strength of acrylic resin polymer reinforced with GF has been shown in studies to increase [14,16].

Fibers of basalt are found in most places across the world. It is an igneous rock, which means it originated as a molten rock. Basalt has long been utilized in architectural tiles and slab casting procedures [38]. Cast basalt liners for steel tubes also have excellent abrasion resistance. Basalt is used as an aggregate in concrete. Mantle decompression melting produces basalt, a typical extrusive volcanic rock. It has big crystals in a delicate quartz matrix. Basalt fibers (BF) are used to replace metal reinforcements like steel and aluminum. Minerals such as pyroxene, plagioclase, and olivine are found in extruded basalt stone. Basalt rock fibers are non-toxic, non-combustible, and non-explosive. The basalt stones are melted at 1400°C to form fibers. Melted basalt matter is stretched into long fibers by a platinum bushing and then pultruded into rebar and basalt mesh.

The purpose of this research is to ascertain how the orientation of the fibers in a heat-cured PMMA resin made using glass and basalt affects the material's flexural strength. Specifically, it was hypothesized that the flexural strength of acrylic denture base resin would not change depending on the orientation of the reinforced fibers.

## Materials and methods

After receiving ethics committee permission (S.No./MPMSU/Academic/2016-17/137), the current study was carried out from September 2017 to October 2018. According to Saleem et al. [38], a specimen size of 30 was determined in each group with a 95% confidence interval, an 80 % power of the test, and an absolute accuracy of 134.5.

Fibers preparation

For the fabrication of reinforced groups in transverse and meshwork patterns, 4-6 mm long and 10% fibers (by weight) [39] were pre-dipped into a polymer and monomer mixture and integrated into the PMMA dough [40]. They were manually put to form a meshwork at the time of packing of heat cure acrylic resin.

Specimens preparation

This analysis included 150 heat-polymerized PMMA samples (Trevalon, Dentsply India Pvt. Ltd., Gurugram, India; pink tone). The original 65x10x3 mm samples were copied onto rectangular acrylic tar using a treated steel pass-on. These metal dies were embedded in an ordinary metal flask (Varsity jar; Jabbar and Company, India) using dental stone (Dentstone, Neelkanth Minichem Pvt. Ltd., Jodhpur, India). When the stone was finally in place, the passes on were removed, and an isolating media (Acralyn-H, Asian Acrylates, Mumbai, India) was slathered on the surface of the stone mold. In order to speed up the setting process, we decided to let the material cool to room temperature. In order to ensure that the PMMA specimens were accurate, we used the manufacturer's specifications (3:1 polymer-to-monomer proportion by volume). Forming and a short curing cycle (74°C for two hours and expanding temperature up to 100°C for 60 minutes) occurred during the dough stage. After being cleaned and repaired, the cups were left to cool in the seats for a while before being de-flasked. Altogether, 150 samples were fabricated using heat-cured acrylic resin; 30 were left unreinforced (Group A), 30 were reinforced with BF in a transverse pattern (Group B), 30 were reinforced with BF in a meshwork pattern (Group C), 30 were reinforced with GF in a transverse pattern (Group D), and 30 were unreinforced (Group E).

All the excess material from the specimen was removed with a tungsten carbide bur. Sandpapers (locally made) of 100, 300, and 500 grits were used, and finally, pumice slurry was used for polishing the specimens and was then buffed with a clean soft dry brush wheel at slow speed.

Thirty specimens of each group were stored underwater for about 21 days in respective beakers at 37°C in an incubator to maintain the temperature. Specimens were collected from the water, dried, and then analyzed in a universal testing machine (Fuel Instruments & Engineers Pvt. Ltd., Kolhapur, Maharashtra, India). The flexural strength was evaluated by applying a load at a cross-head speed of 1.00 mm/min until the specimen broke.

We conducted a thorough analysis of the collected data with SPSS Statistics version 20.0 for Windows (IBM Corp. Released 2011. IBM SPSS Statistics for Windows, Version 20.0. Armonk, NY: IBM Corp.), employing one-way ANOVA and the Tukey-Kramer various correlation test (=0.05).

## Results

Flexural strength was 54.22 MPa in the transverse GF-reinforced test specimens manufactured from heat-cured PMMA; 64.98 MPa in the transverse BF-reinforced test specimens made from heat-cured PMMA; and 46.26 MPa in the unreinforced test specimens (Table 1).

**Table 1:** Mean and standard deviation of flexural strength (MPa) of denture base resins in different groups MPa: megapascal, SD: standard deviation

Test specimen groups	No. of test specimens	Flexural strength (MPa)	
		Mean ± SD	Min-Max
Group A	30	46.26 ± 2.26	43.31-49.79
Group B	30	64.98 ± 1.53	62.65-67.99
Group C	30	76.45 ± 2.67	70.13-79.78
Group D	30	54.22 ± 2.24	51.15-57.80
Group E	30	59.02 ± 2.38	54.82-62.86

Flexural strength was 59.02 MPa for meshwork GF-reinforced test specimens composed of heat-cured PMMA; 76.45 MPa for meshwork BF-reinforced test specimens; and 46.26 MPa for unreinforced test specimens.

The flexural strength was impacted by both the kind of BF and GF reinforcement (F = 768.316, P = 0.001) (Table 2).

**Table 2:** One-way ANOVA test for comparing the different groups of fiber reinforcement in different patterns

Source of variation	Degree of freedom	Sum of squares	Mean square	f-value	p-value
Treatment (between columns)	4	15554	3888.6	768.32	0.0001
Residual (within columns)	145	733.88	5.061		
Total	149	16288			

There was a statistically significant relationship between the flexural strength of the instances and the BF-support (p= 0.0001) (Table 2).

The flexural strength of test samples for the two types of support differs significantly depending on whether they were constructed using a transverse design or a meshwork design (p = 0.001 and p = 0.001 in progression) (Table 3).

**Table 3:** Comparison of flexural strength (MPa) between different reinforcement groups in different patterns using the Tukey-Kramer multiple comparison test ¥ If the value of q is greater than 3.813, then the p-value is less than 0.05 *** highly significant NS: not significant, MPa: megapascal

Comparison	Mean difference	q^¥^	p-value
Group A Vs Group B	-8.72	45.57	0.001***
Group A Vs Group C	-30.19	73.50	0.001***
Group A Vs Group D	-7.96	19.38	0.001***
Group A Vs Group E	-12.76	31.06	0.001***
Group B Vs Group C	-11.47	27.93	0.001***
Group B Vs Group D	10.76	26.19	0.001***
Group B Vs Group E	5.96	14.50	0.001***
Group C Vs Group D	22.23	54.12	0.001***
Group C Vs Group E	17.43	42.44	0.001***
Group D Vs Group E	-4.80	11.68	0.001***

## Discussion

The rising cost of prosthesis repair every year is proof that the problem of broken full dentures is still a challenge for doctors to tackle. Flexural weakness disappointment from cyclic bending manifests as a midline crack at the base of a maxillary or mandibular total dental replacement after protracted usage in vivo [5]. Fibre reinforcing is only successful if the polymer matrix can effectively transfer stress to the fiber [41,42]. The length of the fibers must match or exceed the critical fiber length [41]. Although there may be an optimal fiber length for inhibiting polymer flow, increasing fiber length may change the flow properties of acrylic resin. Depending on the matrix resin, 2 to 4 mm fiber length is recommended for industrial injection moulding [41], and 6 mm is proposed for denture base polymer reinforcement [43-46]. In compression moulding, increasing fiber length did not improve transverse strength or elastic modulus.

In the present study, GF and BF about 4-6 mm in length and 5% by weight were considered in previous studies [13,41-44]. After collection, the samples were kept in beakers of distilled water in an incubator at 37°C for 21 days, the same amount of time used in previous research [41,47-48]. This research looked at the Flexural Strength (FS) of unreinforced and reinforced acrylic resin specimens in two orientations, using the standards set forth by ISO 1567:1999 [49]. This work utilizes the same quantity of GF as other studies [42, 50], that have experimented with GF.

Compared denture base resin and GF arranged perpendicular to the applied force direction and meshwork design [51]. When BF were utilized in a meshwork pattern, flexural strength increased significantly (76.45 2.67 MPa) compared to transversely reinforced acrylic resin specimens with BF (64.98 1.54 MPa). When GF were inserted in a meshwork pattern, flexural strength increased (54.22 2.24 MPa) compared to unreinforced acrylic resin specimens (46.26 2.24 MPa) but not as much as when GF were introduced in a transverse pattern (59.02 2.38 MPa). When comparing the two groups, there was a discernible difference (P = 0.001). This result agrees with what has been found in earlier studies [1,39,52-54].

The current study results indicate that BF were effectively impregnated with polymer matrix resin. Based on these findings, reinforcing denture base resins with BF in both a meshwork and transverse/parallel pattern may be an effective method for enhancing denture base strength beyond normal limits. The highest pressure that has to be coordinated in a certain direction may be handled by BF in a meshwork, making this material ideal for such applications. The polymer grid is supported in two directions by the bidirectional meshwork of basalt filaments, which is useful when it is difficult to predict the direction of the greatest pressure. BF consolidated in a transverse example really built up the polymer grid in only one direction, for example, toward the fibers. As a result, the mechanical qualities of the dental replacement base resin are enhanced by the anisotropic supporting effects of meshwork BF. In this study, the flexural strength of PMMA was determined by testing basalt (both meshwork and transverse) fiber-built and glass (both meshwork and transverse) fiber-supported samples after a water storage timespan of days. This method is clinically relevant because it closely mimics the stacking plan used in actual clinical practice. After 48 weeks of storage in water, Vallittu discovered that test specimens reinforced with E-GF showed a drop in transverse strength of 14% [40]. Most of this depletion happened within the first four weeks of water storage. After three months of storage in water, Lastumaki et al. GF-reinforced composites had a 60% drop in flexural strength [54]. Water may be responsible for leaching ions or oxides from the surface of the GF, which might explain the diminished flexural characteristic. Hydrolytic degradation and the polymer-fiber system may be adversely affected by elements like boron added to E-GF to lower the fibers' high calcium oxide concentration [55]. The flexural strength of basalt (meshwork) fiber-reinforced specimens increased after three weeks in the current investigation. BF and GF composites may be tested for flexural strength after being stored in water for three weeks to three months in future studies.

The most common reason for the failure of dental prosthetics is material fatigue [1,55]. Fatigue failure is caused by tensile stresses developed in specific areas of the prosthesis due to repeated occlusal loads. In spite of a material's ability to withstand occlusal loads at its static strength, fatigue fractures may nonetheless develop [56]. Compared to a non-reinforced dental repair, dynamic In vitro experiments show that GF reinforcement may boost fatigue resistance by a factor of up to 100 [57]. Moreover, the flexural strength of the BF-built instances is much more noticeable than that of the GF-supported ones. Fiber-reinforced composites (FRCs) have been employed for some time in innovations like airplanes that call for high tiredness strength because of claims that they offer exceptional weakness strength [58]. By combining the strengthening action of glass fibers, it is possible to adjust the mechanical qualities of dental prostheses to individual requirements. Using strategically placed BF reinforcement, it is possible to design a prosthesis that can withstand the strains imposed by the masticatory system.

Further research into the incorporation of BF in various orientations to improve the flexural strength of the material is warranted. It is assumed that such incorporated orientation of BF is more favorable for flexural strength improvement and prevention of fracture. But due to the lack of studies and systemic literature in this reference, further in vivo research and control trials are needed to bring it to a proper conclusion. The current in vitro study did not look at other factors that may affect the FRCs, such as salivary presence, salivary pH, or cytotoxicity from oral bacteria. Another restriction is that no investigation of resin material aging or heat cycling was performed. Thermocycling has the potential to improve our understanding of real-world oral circumstances and alter the mechanical characteristics of resin materials. Instead of using only one kind of GF and heat-polymerizing acrylic resin, it would have been better to compare and contrast numerous. Since glass reinforcing has been proven to significantly affect PMMA's water sorption and solubility, it is crucial to study the material's mechanical characteristics, such as flexural strength, after being exposed to long-term water storage in a range of orientations. The use of artificial saliva instead of distilled water for specimen storage will allow for future in vitro experiments that more closely mimic the conditions of the mouth. According to the findings of previous studies [39], it was determined that the glass and carbon fiber-reinforced heat-polymerizing acrylic resin induced mild cytotoxicity by reducing gingival fibroblast proliferation by around 20%.

The study has some limitations. Future studies need to investigate the cytotoxicity of newly created reinforcing fibers in the prosthetic industry, as well as the toxicity of basalt. Understanding the role of periodontal tendons, proprioceptors, complex occlusal contact designs, occlusal stacking, intraoral heat variations, and pH fluctuations will need more randomized clinical pilot studies.

## Conclusions

The following inferences can be made within the scope of the present investigation as different reinforcement materials have their own importance in increasing the strength of denture base PMMA because of their own mechanical property irrespective of direction. Fiber reinforcement in meshwork patterns increases the flexural strength of PMMA more than reinforcement in the transverse pattern. BF reinforcement increases the flexural strength of PMMA more than GF reinforcement.
